# Electromagnetic radiofrequency radiation with special reference to otorhinolaryngology and brain tumors^☆^

**DOI:** 10.1016/j.bjorl.2018.09.003

**Published:** 2018-09-30

**Authors:** Sergei V. Jargin

**Affiliations:** Peoples’ Friendship University of Russia, Moscow, Russia

Dear Editor,

The aim of this letter is to point out the following considerations. (1) There is limited evidence for the carcinogenicity of electromagnetic radiofrequency radiation (ERR) based predominantly on epidemiological studies. However, epidemiological research on radiation risks is associated with bias: dose-dependent selection and self-selection as well as recall bias etc.^1^ (2) There has been no substantial increase in brain tumor incidence despite the tremendous rise of mobile phone use. Animal studies have consistently shown no increase in cancer risk due to long-term exposure to ERR.^2^ (3) The damage per unit of absorbed energy tends to increase with the decreasing wavelength, which is evident not only for ionizing and ultraviolet radiation but also for infrared and visible light causing thermal damage at absorbed energies that would be harmless for ERR heating tissues more evenly, i.e. having a higher penetration depth. (4) Reported risks from ERR are of non-thermal intensity. However, UHF-therapy of thermal intensity has been widely used in the former Soviet Union for the treatment of inflammatory otorhinolaryngolical conditions since the early 1960s, overviewed in.^3^ Associations with cancer have never been reported, although overexposure of tissues such as eye lenses and brain can occur if certain output power levels are exceeded.^4^ Considering anatomical proximity of tonsils, nasal cavity and brain, especially in children, there have been concerns about UHF-therapy (Ultra High Frequency) in otorhinolaryngology. A single case of transitory strabismus and dysphagia in a child coinciding with repeated UHF-therapy for tonsillitis and allergic rhinitis at the age of 4–6 years is known.^3^

ERR are present in the natural environment, fluctuating with the solar activity and atmospheric electricity; it might influence living organisms like the weather does, not necessarily causing harm. ERR may influence neural functions: brain electrical activity, cognitive function, sleep etc.^2^ Transient effects on the brain function and retinal phosphenes are not considered to be adverse health effects, although they can be disturbing.^5^ The same can be said about the association between exposure to ERR and tinnitus, although this relationship has not been well-established.^6^ In conclusion, there is neither compelling evidence nor theoretic plausibility for the concept that ERR is more carcinogenic or causes more structural harm than infrared radiation, which is ubiquitous and harmless up to the thermal damage. However, considering possible functional effects of ERR, precautionary measures recommended by Drs. Medeiros and Sanchez should be agreed with.^6^

## Conflicts of interest

The author declares no conflicts of interest.
